# Quantitative Relationship between Cadmium Uptake and the Kinetics of Phytochelatin Induction by Cadmium in a Marine Diatom

**DOI:** 10.1038/srep35935

**Published:** 2016-10-25

**Authors:** Yun Wu, Zhiqiang Guo, Wei Zhang, Qiaoguo Tan, Li Zhang, Xinlei Ge, Mindong Chen

**Affiliations:** 1 Jiangsu Collaborative Innovation Center of Atmospheric Environment and Equipment Technology (CICAEET), Jiangsu Key Laboratory of Atmospheric Environment Monitoring and Pollution Control (AEMPC), Jiangsu Engineering Technology Research Center of Environmental Cleaning Materials (ECM), School of Environmental Science and Engineering, Nanjing University of Information Science & Technology (NUIST), Nanjing 210044, China; 2 Key Laboratory of Tropical Marine Bio-resources and Ecology, Guangdong Provincial Key Laboratory of Applied Marine Biology, South China Sea Institute of Oceanology, Chinese Academy of Sciences, Guangzhou 510301, China; 3 College of the Environment and Ecology, Xiamen University, Xiamen 361102, China

## Abstract

Heavy metals activate the synthesis of phytochelatins (PCs), while the induced PCs might affect metal uptake via chelating intracellular free metals. However, the relationship of PCs to metal uptake is poorly understood. In this study, we examined the kinetics of cadmium (Cd) accumulation and the synthesis of PCs in a marine diatom, *Thalassiosira weissflogii*, under different irradiance levels. Irradiance alone could not change the concentrations of PCs in the Cd-free treatments, while higher irradiance accelerated the induction of intracellular PCs at the same [Cd^2+^] level. PC-SH (2 × PC_2_ + 3 × PC_3_ + 4 × PC_4_) was bound with Cd at a stoichiometric ratio of 2 to 49 in our short-term uptake experiments, indicating that PC induction is sufficient to serve as the first line of defense against Cd stress. A positive linear correlation between the induction rate of PCs and the Cd uptake rate was observed, while the ratio of the PC content to intracellular Cd varied greatly when the irradiance was increased several fold. Because metal uptake has been successfully used in predicting acute metal toxicity, our findings are helpful for understanding the role of PCs in metal detoxification and developing PCs as biomarkers for metal sensitivity.

Metal contamination in aquatic ecosystems is a global environmental problem. Phytoplankton, which has a considerable biomass, is the entry point for metals into the aquatic food web and is thus important from the perspective of ecotoxicology and human health. Cadmium (Cd) is a priority pollutant that exerts toxicity mainly by nonspecifically binding with the sulfhydryl groups of proteins, displacing the essential metals in metalloenzymes or producing oxidative damage^1^. To deal with the stress due to elevated environmental Cd, phytoplankton may have evolved both extracellular and intracellular strategies to alleviate metal toxicity. It is known that metal uptake can be regulated by decreasing metal internalization by various mechanisms, including the excretion of non-specific ligands, the alteration in the concentration and affinity of metal carriers/channels, and the transformation of metal species^2^,^3^. As a primary adaptive mechanism once metals are inside cells, the metal can be chelated and sequestered in a biologically inactive form, a phytochelatin (PC) complex^4^.

PCs are low molecular weight (LMW) thiols with the general structure (γ-Glu-Cys)_*n*_-Gly (n = 2–11), and PC_2–4_ are the predominant species found in phytoplankton^4^,^5^. PCs are synthesized enzymatically from glutathione (GSH), and Cd is thought to be the strongest activator of PC synthase^6^,^7^. PCs are known as specific intracellular chelators in phytoplankton and respond rapidly to metal exposure^8^,^9^,^10^. PCs sequester metals by chelating metal with the sulfhydryl groups in the PC cysteines. Since their identification thirty years ago, considerable efforts have been made to explore the possibility of using PCs as biomarkers of metal stress^11^,^12^. However, no quantitative relationship has been observed between PC concentration and metal accumulation in previous studies^4^,^13^.

Metal uptake across the cell membrane is driven by an inward diffusion gradient, which is maintained by the rapid binding of intracellular free metal ions to create a non-diffusible form, such as a PC-metal complex. The PC turnover (including synthesis, degradation and excretion) is very fast; therefore, the kinetics may be more crucial than the concentration itself 9,10. Unfortunately, very little work has been conducted on the kinetics of the induction of PCs, let alone the association of these kinetics with metal uptake kinetics.

As an important environmental factor, irradiance has a great impact on the physiological and biochemical processes of phytoplankton. Miao and Wang^14^ found that increased irradiance could enhance Cd uptake by the diatom *Thalassiosira pseudonana*. It has also been proposed that nitrogen (N) uptake and accumulation are greatly dependent on irradiance^15^,^16^. Changes in the substrate (N) and activator (Cd) caused by irradiance may potentially affect amino acids and subsequently the synthesis of peptides (PCs). To our knowledge, there has been no study of the effects of irradiance on PC induction. Therefore, irradiance was used in this study to modify Cd uptake by the most studied coastal diatom, *Thalassiosira weissflogii*. The corresponding synthesis of PCs during a 4-h exposure period was quantified to investigate how irradiance influences PC induction. Afterward, changes in the PC induction rate and metal uptake rate were investigated with a regression analysis to determine whether there was any correlation between Cd uptake and the kinetics of PC induction by Cd.

## Materials and Methods

### Diatom cultures

The stock of *Thalassiosira weissflogii* (CCMP 1587), obtained from Provasoli-Guillard National Center for Marine Algae and Microbiota, Bigelow Laboratory, was maintained in f/2 medium^17^ in a growth incubator (23 ± 1 °C, 120 μmol photons m^−2^ s^−1^, under a 14-h light:10-h dark regimen). Throughout this study, 0.22 μm-filtered and chelexed (with Chelex 100 resin, Bio-Rad Laboratories, CA) Aquil artificial seawater (containing NaCl, KBr, KCl, H_3_BO_3_, Na_2_SO_4_, NaHCO_3_, NaF, CaCl_2_, SrCl_2_ and MgCl_2_, and without EDTA, trace metals or vitamins)^18^ was used. Stocks for the f/2 medium (N, P, Si and vitamins) were also passed through the column with Chelex 100 ion-exchange resin to remove any background metals. The pH value of the medium was maintained at 8.2 ± 0.1. All the polycarbonate flasks and bottles were soaked in 5% HCl for at least 24 h and rinsed seven times with Milli-Q water (18.2 MΩ) before the experiments.

### Uptake kinetics

*T. weissflogii* cells were first transferred from the f/2 culture medium to f/2-f/20 medium (with f/20 levels of trace metals without EDTA or the addition of Cu and Zn) and acclimated at three levels of irradiance (60, 120 and 240 μmol photons m^−2^ s^−1^) for two weeks. Irradiance levels ranging from 30 to 300 μmol photons m^−2^ s^−1^ are usually employed for marine algae in the laboratory, although these levels are much higher than those in the field^19^. The temperature and light-dark cycle conditions were the same as those of the stock culture. During the acclimation period, the cell density was measured daily with a hemocytometer, and the culture medium was renewed every 3–5 days to ensure exponential growth of the cells. Afterward, a 4-h uptake experiment was conducted on the irradiance-conditioned cells. Mid-log growing diatoms were harvested by centrifugation (3000 rpm, 23 °C, 10 min) and resuspended in 250 mL of pre-equilibrated Cd uptake medium. Seawater not enriched with nutrients was employed for the uptake experiments. There were four Cd-concentration treatments with two replicates each in every irradiance test, and the total dissolved Cd concentrations (added as a certified Cd ICP standard solution, 1000 mg L^−1^, NSI solutions) were 2.7 × 10^−8^, 8.9 × 10^−8^, 2.7 × 10^−7^, and 4.5 × 10^−7^ mol L^−1^. Nitrilotriacetate (NTA, 0.1 mM) was added to maintain a constant free Cd ion concentration ([Cd^2+^]). After the addition of ultrapure NaOH (1 M) to adjust the pH (8.2 ± 0.1), the uptake medium was equilibrated overnight before use. The calculated [Cd^2+^] (obtained using the Visual MINTEQ software, version 3.1) for each corresponding treatment was 5.49 × 10^−10^, 1.83 × 10^−9^, 5.50 × 10^−9^, and 9.17 × 10^−9^ mol L^−1^, respectively.

The uptake of Cd into the diatoms was studied under three irradiance levels as described for the acclimation period. At 1, 2, 3 and 4 h of exposure, the intracellular Cd concentration was measured in each treatment and the (net) Cd uptake rate was calculated from the slope of the linear regression between the intracellular Cd concentrations and time. For this measurement, a 20-mL sample from each replicate bottle was gently filtered (<50 mmHg) through a 3-μm polycarbonate membrane, rinsed three times with artificial seawater, and then resuspended in 10 mL of EDTA (0.1 mM) to remove the weakly surface-adsorbed Cd. The cells were collected again and oven-dried at 60 °C for 24 h and then digested in 1 mL of ultrapure concentrated nitric acid (Fisher Scientific, USA) overnight, followed by dilution with Milli-Q water. The Cd samples were finally analyzed by inductively coupled plasma mass spectrometry (ICP-MS, Agilent 7700) with internal standards. Additionally, a “control” treatment with irradiance acclimation but without Cd exposure was prepared to quantify Cd and thiols, and the Cd content of this treatment fell below the detection limit (0.02 ppb, 1.8 amol Cd cell^−1^). The number of cells at the beginning and end of the uptake experiments was counted with a microscope (at approximately 3–8 × 10^4^ cells mL^−1^), and all the concentrations in this study were expressed on a per cell basis.

### Measurement of LMW thiols

In tandem with the Cd uptake sampling, the diatom culture was also harvested for the determination of LMW thiols (cysteine, Cys; γ-glutamylcysteine, γ-EC; GSH; PC_2–4_). A 200-mL aliquot was concentrated, rinsed three times with filtered artificial seawater, and then stored in 1 mL of an ice-cold 6.3 mM diethylenetriamine pentaacetic acid (DTPA) with 0.1% (v/v) trifluoroacetic acid (TFA) extraction solution. Afterward, algal cells were ultrasonically disrupted (6 times, 10 sec each time with 10 sec intervals), with a breakage efficiency of over 95%. The homogenate was then centrifuged at 13,000 *g* for 10 min (4 °C), and the separated supernatant was subjected to pre-column derivatization as described in previous studies^20^,^21^. Briefly, 250 μL of supernatant was added to a disulfur reductant consisting of 615 μL of 4-(2-hydroxyethyl)-piperazine-1-propane-sulfonic acid (HEPPS, 200 mM in 6.3 mM DTPA, pH 8.2) and 25 μL of tris (2-carboxyethyl) phosphine hydrochloride (TCEP, 20 mM in HEPPS buffer). A volume of 10 μL of *N*-acetyl-L-cysteine (NAC, 0.5 mM) was also added as an internal standard to trace the HPLC performance. The mixture was placed in a 45 °C water bath for 10 min, and later labeled with 10 μL of the fluorescent dye monobromobimane (mBBr, 50 mM). After incubating at 45 °C in a water bath for another 30 min, these reactions were ceased by adding 100 μL of methanesulfonic acid (MSA, 1 M). Simultaneously, a series of standard mixtures (with Cys, GSH and γ-EC at concentrations of 4, 8, 16, 32 and 40 pmol μL^−1^ added and PC_2–4_ at concentrations of 0.8, 1.6, 3.2, 6.4 and 8.0 pmol μL^−1^ added) were treated in the same way to aid in quantification. All the chemicals used in this study were of the highest purity available. The levels of LMW thiols were finally determined by a gradient elution method^22^ using an HPLC instrument (Thermo U-3000) equipped with a Phenomenex Synergi 4 μ Hydro-RP C_18_ column (100 × 4.6 mm) and an online FLD-3100 fluorescence detector. The (net) induction rate (*I*, amol cell^−1^ h^−1^) of PC thiols was calculated as the time-weighted average of *C* according to the following equation^23^:


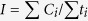


where *t*_*i*_ is the time of exposure (h), and *C*_*i*_ is the concentration of the newly induced thiols after *t*_*i*_.

### Statistical analysis

Differences among treatments were evaluated using a one-way ANOVA with Duncan’s post hoc multiple comparisons test (SPSS 16.0 software). The differences were considered significant when *p* < 0.05.

## Results

### Cd uptake

**Figure 1:**
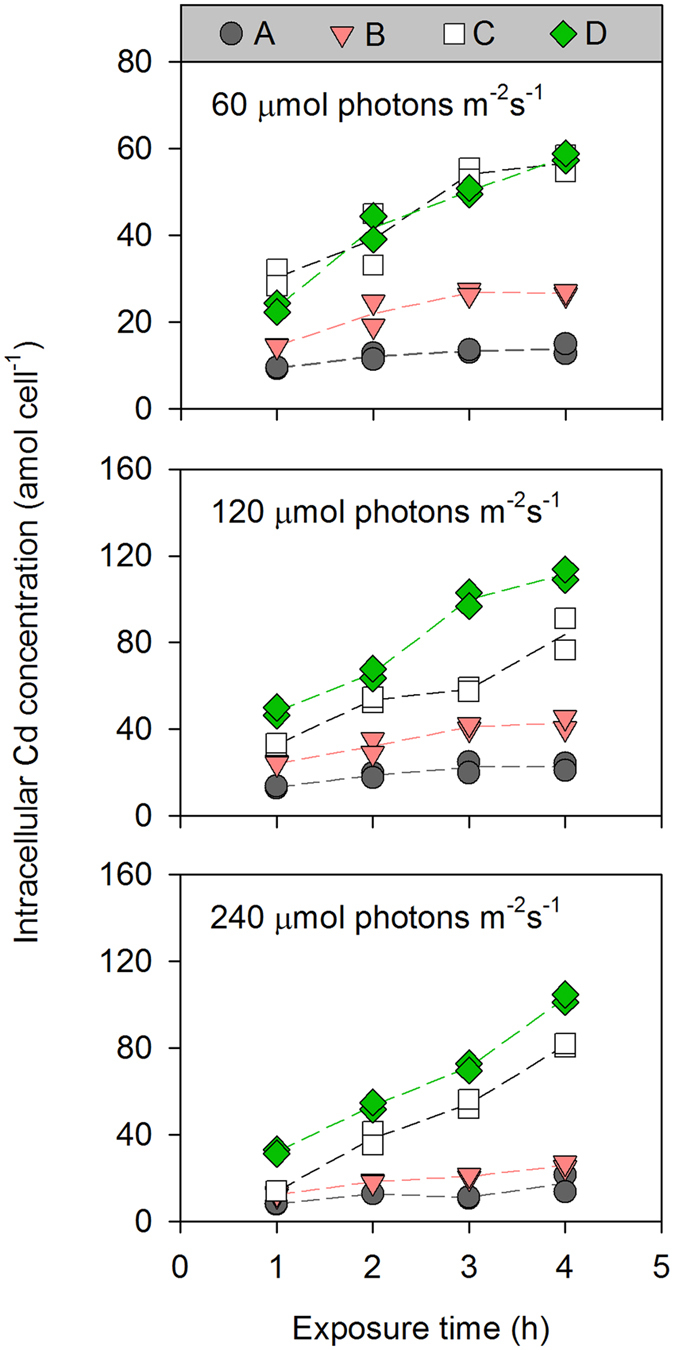
Uptake of Cd by the diatom *Thalassiosira weissflogii* maintained at different irradiance levels. The constant free Cd ion concentrations ([Cd^2+^]) in treatments (**A–D**) were 5.49 × 10^−10^, 1.83 × 10^−9^, 5.50 × 10^−9^, and 9.17 × 10^−9^ mol L^−1^, respectively. Each dot is one replicate (*n* = 2), solid lines represent the trends of their mean values.

**Figure 2:**
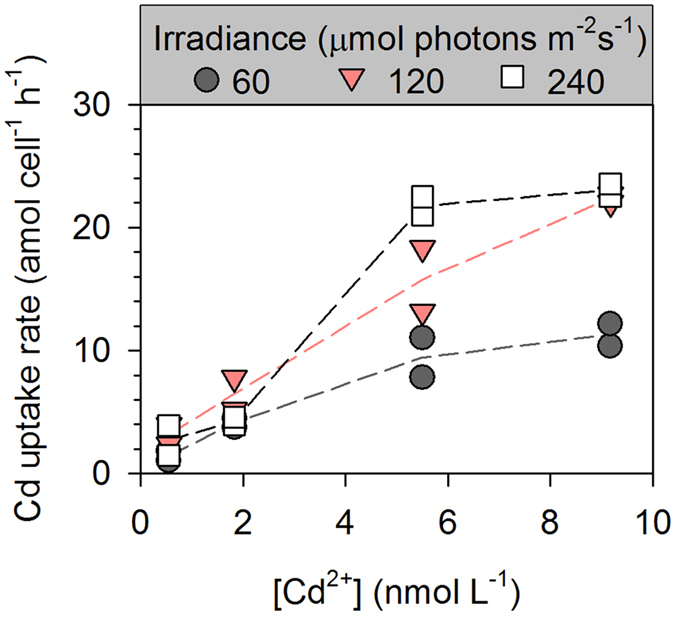
Cd uptake rates in the diatom *T. weissflogii* as a function of the constant free Cd ion concentrations ([Cd^2+^]). The uptake rate was calculated from the slope of the linear relationship between the intracellular Cd concentration and the exposure time. Each dot is one replicate (*n* = 2), solid lines represent the trends of their mean values.

Generally, a linear relationship between the intracellular Cd concentration and the exposure time was observed for all irradiance and [Cd^2+^] treatments (Fig. 1), and the slope of the linear regression was calculated to obtain the Cd uptake rate, which was plotted as a function of dissolved [Cd^2+^] (Fig. 2). It was obvious that the Cd uptake rate of *T. weissflogii* increased with [Cd^2+^] in all the irradiance treatments, whereas the influence of irradiance on Cd uptake changed with increasing [Cd^2+^]. At a [Cd^2+^] ≤ 1.83 × 10^−9^ mol L^−1^, the Cd uptake rate was comparable among the three irradiance treatments (2.43 ± 1.24 amol cell^−1^ h^−1^ for 5.49 × 10^−10^ mol L^−1^ and 4.97 ± 1.47 amol cell^−1^ h^−1^ for 1.83 × 10^−9^ mol L^−1^). At higher levels of [Cd^2+^], an obvious increase in the Cd uptake rate was observed at the high-irradiance level, reaching up to 23.05 ± 0.50 amol cell^−1^ h^−1^ (the means ± semi-range) at the highest [Cd^2+^].

### Kinetics of thiol compounds induction

**Figure 3:**
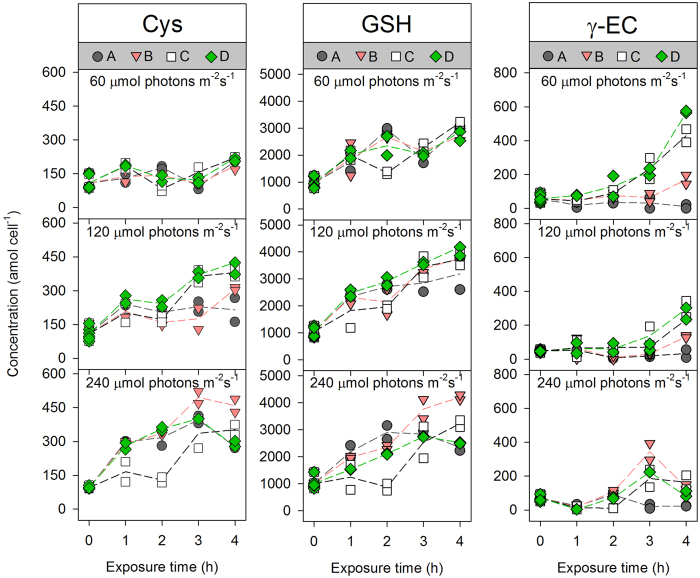
Kinetics of the induction of phytochelatin precursors (Cys: left; GSH: middle; γ-EC: right) in *T. weissflogii* maintained at different irradiance levels. The constant free Cd ion concentrations ([Cd^2+^]) in treatments (**A–D**) were 5.49 × 10^−10^, 1.83 × 10^−9^, 5.50 × 10^−9^, and 9.17 × 10^−9^ mol L^−1^, respectively. Each dot is one replicate (*n* = 2), solid lines represent the trends of their mean values.

**Figure 4:**
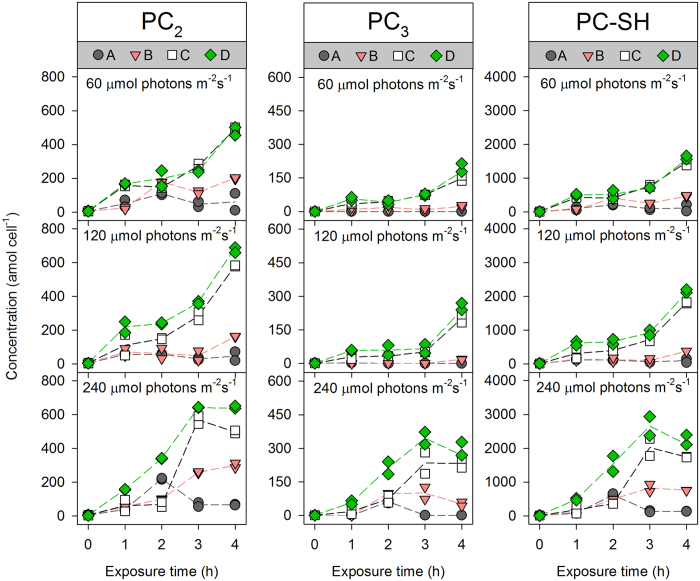
Kinetics of phytochelatin induction (PC_2_: left; PC_3_: middle; PC-SH = 2 × PC_2_ + 3 × PC_3_ + 4 × PC_4_: right) in *T. weissflogii* maintained at different irradiance levels. The constant free Cd ion concentrations ([Cd^2+^]) in treatments (**A**–**D**) were 5.49 × 10^−10^, 1.83 × 10^−9^, 5.50 × 10^−9^, and 9.17 × 10^−9^ mol L^−1^, respectively. Each dot is one replicate (*n* = 2), solid lines represent the trends of their mean values.

Without Cd exposure, the variation in the concentrations of thiol compounds was small among the control treatments (0 h in Figs 3 and 4), suggesting that irradiance did not significantly affect PC synthesis in diatoms. With an increase in the [Cd^2+^], Cd induced different patterns in the synthesis of thiol compounds in *T. weissflogii* at different irradiances. Of the five thiol compounds, GSH was always at the highest concentration, while PC_3_ was generally at the lowest concentration. As the dominant thiol compound, GSH remained relatively stable in the algal cells and increased 2.3–4.2 fold after 4 h of Cd uptake compared with the control (middle panel in Fig. 3). Similarly, the content of Cys fluctuated and exhibited a slight increase at the end of the exposure period (left panel in Fig. 3). Regarding γ-EC, the content of this thiol compound generally exhibited a lag phase followed by rapid increases after 2 h of Cd exposure. Additionally, at the lowest irradiance and highest Cd level tested, the γ-EC contents increased ~10-fold compared to the pre-exposure levels (right panel in Fig. 3). In contrast with the PC precursors, PC_2_ and PC_3_ responded rapidly at the lowest [Cd^2+^] and irradiance levels; within 1 h of exposure, the contents of these PCs increased from 4.8 to 57.1 ± 3.4 amol cell^−1^ for PC_2_ and from 0.6 to 2.5 ± 1.1 amol cell^−1^ for PC_3_. These increments were maintained with increasing exposure time, dissolved [Cd^2+^], and irradiance levels (Fig. 4). At the highest irradiance level, a plateau or even a decrease was observed between 3–4 h for both PC_2_ and PC_3_, accompanied by the emergence of PC_4_ (data not shown). Therefore, the calculated PC-SH (2 × PC_2_ + 3 × PC_3_ + 4 × PC_4_) in *T. weissflogii* revealed a pattern similar to, but more pronounced than, that of PC_2_ and PC_3_ (right panel in Fig. 4).

**Figure 5:**
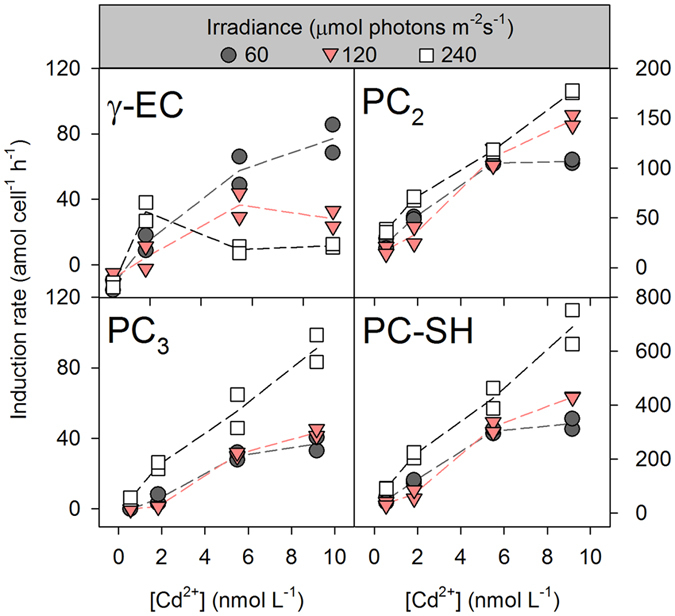
Phytochelatin induction rates (PC-SH = 2 × PC_2_ + 3 × PC_3_ + 4 × PC_4_) in the diatom *T. weissflogii* as a function of the constant free Cd ion concentrations ([Cd^2+^]). The induction rate was calculated as the time-weighted average of the concentration of intracellular thiols with exposure time. Each dot is one replicate (*n* = 2), solid lines represent the trends of their mean values.

Because the intracellular concentrations of the four thiol species (γ-EC, PC_2_, PC_3_ and PC-SH) increased non-linearly with exposure time, we estimated the induction rate of these thiols using time-weighted values. It was clear that the induction rate of most thiol species increased with increasing [Cd^2+^], but the pattern was dependent on the irradiance level (Fig. 5). At the lowest [Cd^2+^], there was no significant difference in the induction rate of γ-EC among the three irradiance treatments (*p* > 0.05). At a higher [Cd^2+^], the increase in the γ-EC induction rate was more precipitous in the low irradiance treatment. For example, at a [Cd^2+^] of 9.17 × 10^−9^ mol L^−1^, the γ-EC induction rate decreased significantly from 77.0 ± 8.5 amol cell^−1^ h^−1^ at 60 μmol photons m^−2^ s^−1^ to 11.6 ± 1.0 amol cell^−1^ h^−1^ at 240 μmol photons m^−2^ s^−1^. In contrast, the induction rates of both PC_2_ and PC_3_ increased with increasing irradiance, especially when the irradiance increased from 120 to 240 μmol photons m^−2^ s^−1^. Consequently, the induction rate of the mixed PC-SH was significantly higher at the high irradiance level than at the lower irradiance levels.

### Data analysis

**Figure 6:**
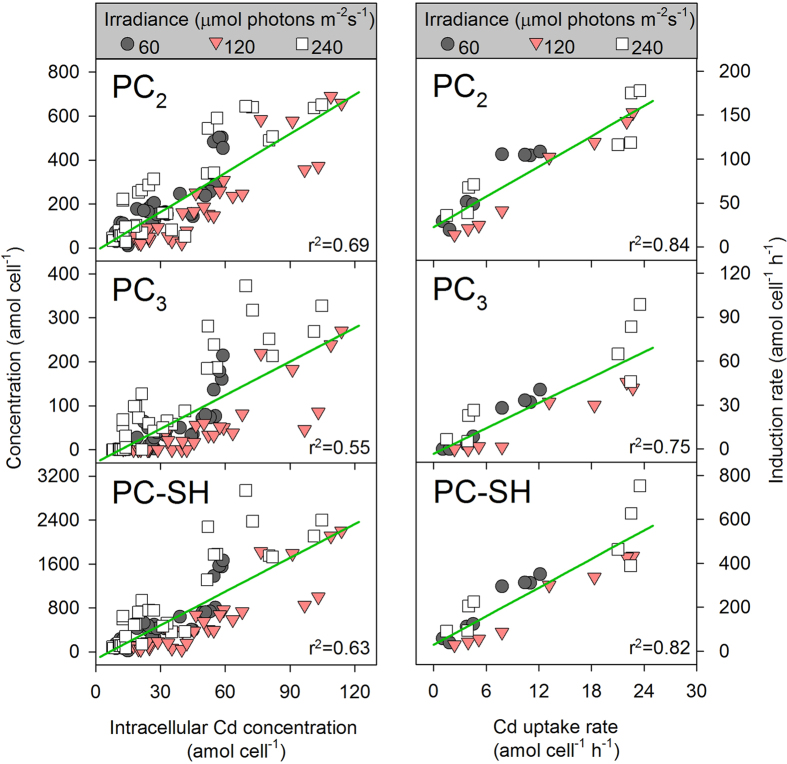
Left panel: Phytochelatin concentrations as a function of intracellular Cd concentration. The solid lines represent the linear regression of the phytochelatin concentration and Cd concentration. Right panel: Phytochelatin induction rates (PC-SH = 2 × PC_2_ + 3 × PC_3_ + 4 × PC_4_) as a function of the Cd uptake rate. The solid lines represent the linear regression of the phytochelatin induction rate and the Cd uptake rate. Each dot is one replicate (*n* = 2).

The changes induced in the concentrations of PCs as a function of intracellular Cd are shown in Fig. 6. Although the correlation between the three thiol concentrations and the Cd concentrations were significant, the *r*^2^ values were generally lower. Given our findings that the PC induction rate and Cd uptake rate exhibited the same trends along with variations in the irradiance and [Cd^2+^], the relationship of these rates was further analyzed by a linear regression (Fig. 6). There was a strong correlation between each thiol induction rate and the Cd uptake rate (*p* < 0.001), indicating that the kinetics of PC induction was closely related to intracellular Cd uptake.

## Discussion

In this study, irradiance was demonstrated to have great effects on the uptake of Cd by *T. weissflogii*. Increased irradiance could enhance Cd uptake by the cells, especially when the [Cd^2+^] was high (Fig. 2). Such effects of irradiance have also been found in many other diatom species after short-term (4 h) or long-term (72 h) Cd exposure^14^,^24^. Herein, we propose several hypotheses in an attempt to explain the observed effect. First, light irradiance may affect the biochemical composition and physiological status of marine phytoplankton (e.g., the C:N ratio, the pigment content and ratio, and photosynthesis)^25^,^26^,^27^. For example, more N uptake at a high level of irradiance may lead to the synthesis of more ligands for Cd transport and the induction of more chelators (e.g., PCs) for binding Cd internally. Second, the metal uptake rate has been proven to be dependent on the growth rate of diatoms^14^. Under high irradiance levels, the metal uptake rate increased proportionally with the increase in cell growth to maintain a constant cellular metal content. Third, in the Zn-limited cultures (Zn was removed by the Chelex-100 resin in this study), Cd could partially substitute for Zn and serve as a metal cofactor in cadmium carbonic anhydrase, which plays a vital role in the acquisition of inorganic carbon in diatoms^28^. Thus, the physiological requirement for Zn may increase Cd uptake to sustain normal cellular activity. However, whether these explanations reveal the essential mechanisms or other more plausible explanations exist must to be further examined. It should be noted that Cd uptake represented the difference between influx and efflux in our short-term uptake experiments (4 h), despite the fact that metal efflux may be considered negligible compared with the much higher metal influx^2^.

Consistent with our previous study^10^, both PC_2_ (4.8 amol cell^−1^) and PC_3_ (0.6 amol cell^−1^), which are essential for cells to adapt to environmental fluctuations in metals, were detected in the control algae. Moreover, there was no significant difference in the background PC content among the three irradiance treatments, implying that irradiance could not effectively induce the synthesis of PCs. Previous studies have also demonstrated that PCs are specifically induced by heavy metals, whereas other treatments, such as heat/cold shock, altered hormonal levels, oxidative stress and anoxia, did not result in the induction of PCs in cell culture^8^. PCs are synthesized enzymatically by PC synthase, which is present inherently in plant and algal cells^6^,^29^. This preexisting enzyme system is responsible for a rapid response to Cd exposure; a significant synthesis of PC_2_ and PC_3_ was observed within 1 h at the lowest [Cd^2+^] and irradiance level. When we combined our data from this study with the results from previous studies^9^,^10^, we found that the induction of PC_2_ and PC_3_ within 4 h of Cd exposure was much more obvious and rapid than that under Pb or Hg stress. In addition, the induction of PC_4_ was detectable after a 3-h exposure to pCd 8.0 (9.17 × 10^−9^ mol L^−1^), more rapid than that after exposure to pPb 7.1 for 4 h or to pHg 6.0 for 12 h. These results demonstrate again that the induction capability of Cd is much greater than that of other metals. Although irradiance by itself did not induce PC synthesis, light could affect PC induction under Cd stress. At the same [Cd^2+^] level, the highest PC_2_, PC_3_ and PC_4_ induction rates or concentrations occurred in the high irradiance treatment. From the perspective of substrate, N uptake and assimilation have been proposed to be dependent on light^15^,^16^. Because N is a necessary element for amino acid and peptide synthesis, more cysteine and PCs might be produced with the higher level of cellular N at high irradiance after Cd exposure. Additionally, the greater rates of γ-EC production at lower light levels may reflect a greater sink for γ-EC due to the production of PCs at higher light levels. From the perspective of an activator, the diatoms accumulated more Cd under higher irradiance as discussed above, thus leading to higher and more rapid PC synthesis in the algal cells.

During short-term exposure to Cd, GSH was synthesized from γ-EC and then consumed for PC synthesis. In the highest [Cd^2+^] treatment, more consumption by PCs and a lower γ-EC supply produced a lower level of GSH at the highest irradiance. Generally, a slight oscillation was observed in the GSH content under stress conditions, similar to the results from many previous studies^9^,^30^,^31^. In addition to chelation, GSH has many other functions in phytoplankton, including antioxidation, catalysis, and amino acid transport^32^. To adapt to a changing environment, it is thus critical for algae to tightly regulate the GSH content.

The levels of [Cd^2+^] used in this study were slightly higher than the reported no-observed-effect concentrations (NOEC) based on toxicity parameters (i.e., cell-specific growth rate or pulse-amplitude-modulated parameters) over a 96-h period^33^. To alleviate the toxic effects caused by Cd, PCs were rapidly induced and chelated the intracellular free metal ions. Theoretically, the PC-SH contents should keep pace with the increase in the concentration of accumulated Cd, and a constant ratio of PC-SH to intracellular Cd would then be expected. However, this ratio varied greatly (2–49) at different irradiances, and a low *r*^2^ value was observed after a linear regression analysis. In addition to environmental factors, the exposure time also had a strong impact on the PC-SH/intracellular Cd ratio. During the 4-h (short-term) exposure period in our study, this ratio was greater than 4 in most cases, while the intracellular Cd concentration exceeded the PC-SH concentration by many fold after a long-term exposure to similar levels of Cd^4^,^13^. Le Faucheur *et al.*^34^ proposed that the stoichiometry of thiol groups binding with Cd was 4, or at least 2. These observations suggest that PC induction is sufficient to remain the first line of defense against Cd stress; however, PC induction appears to be less important in chelation after prolonged metal exposure. Such temporal variation can be explained by the fact that PCs have a rapid turnover rate^9^,^10^ and that more highly polymerized PCs are induced as the exposure period increases^35^. Therefore, using the PC-SH concentration alone might underestimate the contribution of PCs to metal detoxification. The kinetics of PC induction, rather than the concentration of PCs, might be more useful to indicate biologic responses as well as to disclose the relationship between PC induction and metal accumulation.

Based on the facts described in this study and previous studies, the following can be stated: (1) the induction of PCs is a metal specific response and does not result from other environmental stresses; (2) the greatest amount of intracellular Cd was found in the heat-stable protein subcellular fraction, in which the chelated PC-metal complexes might also exist^24^,^36^; (3) the synthesis, transportation, degradation and excretion of PCs is very fast^9^,^10^; (4) both the Cd uptake rate and PC induction rate increased with irradiance, and herein, we have postulated for the first time that Cd uptake may be more closely related to the kinetics of PC induction by Cd than to PC levels, in marine phytoplankton. As expected, a good linear regression was observed between the Cd uptake rate and the induction rate of each PC, with higher r^2^ values than that of the linear regression between the concentrations of Cd and PCs. These results indicate that the kinetics of PC induction and intracellular metal uptake are strongly coupled. The initiation of the synthesis of PCs can only be activated by heavy metal stress, and the PC-metal complexes that form may then be transported into vacuoles or excreted to the environment^2^,^37^, resulting in reduced intracellular free metal ion concentrations. Because protein carriers and major ion channels provide the major means by which hydrophilic metal species cross the cell membrane, changes in the concentration gradient of metal ions caused by PC complexation will in turn affect the uptake of metal across the cell membrane. Therefore, the turnover of PCs (measured as kinetics) may correlate more closely to the changing intracellular metal content than does the absolute concentration of PCs. A few previous studies also reported that the ratio of PC-SH to intracellular metal changed during short-term Pb or Hg exposure^9^,^10^,^38^. However, to date, there has been relatively little research conducted on the kinetics of PC induction, including the association of PC induction kinetics with metal accumulation. It is thus necessary to verify our results using other organisms or metals.

The relationship between the metal uptake rate and the PC induction rate has a significant implication regarding metal toxicity. Since they were identified thirty years ago^5^, PCs have been expected to be useful as biomarkers of environmental metal pollution. In both laboratory experiments and field studies, elevated levels of PCs have been detected as a response to metal stress^11^,^12^, but a quantitative relationship between the concentration of PCs and metal sensitivity was difficult to ascertain^39^. Furthermore, the chelation ratio of PC-SH to intracellular metal also cannot be used to indicate metal toxicity under all conditions^40^. Considering the specific induction of PCs and the rapid turnover rate of these thiols, we hypothesize that PC kinetics is possibly related to metal sensitivity in marine phytoplankton. Recently, Tan and Wang^41^ developed the biotic ligand model (BLM) and used the metal uptake rate to predict the acute toxicity of Cd in small invertebrates (*Daphnia*). These authors proposed that biotic ligands were primarily carriers or channels of cations on cell surfaces; the metal uptake rate should thus be proportional to the concentration of metal binding to the biotic ligands, which was the most relevant predictor of metal toxicity according to the assumptions of the BLM. Our present study provided the first experimental evidence to quantify the link of PC-SH to metal uptake. To better understand the role of PCs in detoxification and utilize PCs as biomarkers, the PC kinetics-metal uptake-metal sensitivity relationship must be directly tested using quantitative experiments.

To conclude, irradiance did not itself induce PC synthesis in marine phytoplankton. Under Cd stress, however, higher irradiance increased the induction of intracellular PCs. This irradiance effect may be partly explained by a greater uptake of Cd or higher substrate accumulation in the cells. Moreover, a strikingly linear association between the PC induction rate and Cd uptake rate was observed when the irradiance varied from 60 to 240 μmol photons m^−2^ s^−1^. We have also demonstrated that dynamic changes of the induction of PCs were more closely related than the static PC concentrations to the accumulated Cd. PC kinetics may be further applied to studying the toxic effects of heavy metals, especially because metal uptake has been successfully used in predicting metal toxicity. In the future, additional work with a wider range of species will be required to test our hypothesis.

## Additional Information

**How to cite this article**: Wu, Y. *et al.* Quantitative Relationship between Cadmium Uptake and the Kinetics of Phytochelatin Induction by Cadmium in a Marine Diatom. *Sci. Rep.* **6**, 35935; doi: 10.1038/srep35935 (2016).
